# Health worker attrition at a rural district hospital in Rwanda: a need for improved placement and retention strategies

**DOI:** 10.11604/pamj.2017.27.168.11943

**Published:** 2017-07-04

**Authors:** Jackline Odhiambo, Felix Cyamatare Rwabukwisi, Christian Rusangwa, Vincent Rusanganwa, Lisa Ruth Hirschhorn, Evrard Nahimana, Patient Ngamije, Bethany Lynn Hedt-Gauthier

**Affiliations:** 1 Partners In Health/Inshuti Mu Buzima, Kigali, Rwanda; 2 Ministry of Health, Kigali, Rwanda; 3 Department of Global Health and Social Medicine, Harvard Medical School, Boston, USA; 4 Partners In Health, Boston, USA; 5 Ariadne Labs, Boston, USA

**Keywords:** Human resources for health, health workforce, retention, compulsory service, turnover, Africa

## Abstract

**Introduction:**

The shortage and maldistribution of health care workers in sub-Saharan Africa is a major concern for rural health facilities. Rural areas have 63% of sub-Saharan Africa population but only 37% of its doctors. Although attrition of health care workers is implicated in the human resources for health crisis in the rural settings, few studies report attrition rates and risk factors for attrition in rural district hospitals in sub-Saharan Africa.

**Methods:**

We assessed attrition of health care workers at a Kirehe District Hospital in rural Rwanda. We included all hospital staff employed as of January 1, 2013 in this retrospective cohort study. We report the proportion of staff that left employment during 2013, and used a logistic regression to assess individual characteristics associated with attrition.

**Results:**

Of the 142 staff employed at Kirehe District Hospital at the start of 2013, 31.7% (n=45) of all staff and 81.8% (n=9) of doctors left employment in 2013. Being a doctor (OR=10.0, 95% CI: 1.9-52.1, p=0.006) and having up to two years of experience at the hospital (OR=5.3, 95% CI: 1.3-21.7, p=0.022) were associated with attrition.

**Conclusion:**

Kirehe District Hospital experienced high attrition rates in 2013, particularly among doctors. Opportunities for further training through Rwanda’s Human Resources for Health program in 2013 and a two-year compulsory service program for doctors that is not linked to interventions for rural retention may have driven these patterns. Efforts to link these programs with rural placement and retention strategies are recommended.

## Introduction

Developing countries, particularly in rural areas, suffer from a shortage and uneven distribution of human resources for health [1–4]. Globally, 50% of the population resides in rural settings but roughly 25% of doctors and 38% of nurses work in these areas [5]. Sub-Saharan Africa, which bears a quarter of the global disease burden [4] and has 63% of her population living in rural settings [6], only has 3% of the world’s trained health workforce [4] with 37% of doctors and 49% of nurses and midwives working in the rural areas [7]. The same pattern exists in Rwanda where with 82% of the population residing rurally, only 12% of physicians and 42% of nurses serve in rural areas [8]. This dearth and disparity in the distribution of health care workers exacerbates health inequities in the rural areas [8–10].

Health care worker attrition is a large driver of the rural human resources for health crisis [5]. In 2010, the World Health Organization recommended policies for attraction, recruitment and retention of health care workers in the rural settings [5]. Recommendations included recruitment of health care workers with a rural background, inclusion of rural health needs in training curricula, and use of mandatory rural service [5]. Rwanda implemented some of these initiatives including a compulsory service program for newly graduate doctors that began in 2010, and required two years of service at district hospitals after receiving medical practice license.

Rwanda’s third health sector strategic plan outlined initiatives to reduce the overall shortage of health workers, as well as improve their quality [11]. Improvement in management, rational deployment, equitable distribution and retention of health care workers were identified as necessary steps to ensure health workers were adequately available in the rural settings [11]. In 2012, through the Human Resources for Health program, Rwanda began the implementation of a national health worker training program to improve the quantity, diversity and quality of health workers [9]. The Human Resources for Health program enrolled doctors, many of whom left rural hospitals to join residency programs in urban settings for specialty training. With little known about health worker attrition in rural Rwanda, our study evaluated the rates and individual factors associated with attrition at a rural district hospital in the setting of a two-year compulsory service and the Human Resources for Health program.

## Methods

*Study design, setting and participants:* We conducted this retrospective cohort study at Kirehe District Hospital, a public facility located in the Eastern Province of Rwanda. Kirehe Hospital is managed by the Ministry of Health with support from an international non-governmental organization, Partners In Health. Kirehe, like other Rwanda Ministry of Health district hospitals, receives graduate doctors annually through the compulsory service program. The study included all salaried healthcare workers, including compulsory service program doctors, employed at Kirehe Hospital on January 1, 2013. Volunteers, interns, daily hires and administrative staff were excluded.

*Data collection and analysis:* We retrieved attrition and individual characteristics data from human resources files, payrolls, contracts and employee’s curriculum vitae. We described the study population and rates of attrition, defined as leaving employment any time during 2013, stratified by type of staff and staff demographics. Because of the use of routinely collected data, data on some variables were unavailable for some staff; in such cases, we reported the reduced sample size. We used Fisher’s exact test to assess the binary relationship of the individual characteristics and attrition reporting p-values. In the multivariate analysis, we used backward stepwise logistic regression to identify potential factors related with attrition, stopping when remaining factors were significant at the α=0.05 significance level. Stata v12.1 (StataCorp, 2012, College Station, TX, USA) was used for analyses.

*Ethics:* the study received ethical approval from the Rwanda National Ethics Committee (IRB 00001497) and Brigham and Women’s Hospital IRB (2009-P-001941/12; BWH) in Boston, USA. A form linking study identifiers to individual identifiers was kept separately from data forms and destroyed after data validation. Consent for participation was not applicable.

## Results

As of January 1 2013, there were 142 salaried health care workers at Kirehe District Hospital (Table 1). Doctors represented 7.7% (n=11) of the staff and 60.6% (n=86) were nurses and midwives. The majority of the staff were male (54.9%, n=78) and in time-bound contracts (65.4%, n=66/101), that is contracts for a specified period of time (65.4%, n=66/101). Nearly half (48.4%, n=62/128) had only secondary level of education, 43.3% (n=61/141) were aged 20-30 years and 46.9% (n=53/113) had worked in the hospital for up to two years.

**Table 1:** Characteristics and bivariate analysis of attrition of health workers

	All health workers N=142 (100%)		Retained N=97 (68.3%)		Left employment N=45 (31.7%)		
	n	%^††^	n	%^‡‡^	n	%^‡‡^	p-value
**Type of health care worker**							
Doctors	11	7.7	2	18.2	9	81.8	0.001
Nurses & Midwives	86	60.6	60	69.8	26	30.2	
Clinical support staff^†^	13	9.2	8	61.5	5	38.5	
Non-clinical support staff^‡^	32	22.5	27	84.3	5	15.6	
**Sex**							
Female	64	45.1	45	70.3	19	29.7	0.718
Male	78	54.9	52	66.7	26	33.3	
**Age group**	N=141						
20-30 years	61	43.3	37	60.7	24	39.3	0.187
31-40 years	60	42.5	44	73.3	16	26.7	
>40 years	20	14.2	16	80.0	4	20.0	
**Education level**	N=128						
Secondary	62	48.4	38	61.3	24	38.7	0.048
Two year university	42	32.8	33	78.6	9	21.4	
Bachelors	24	18.8	12	50.0	12	50.0	
**Marital status**	N=131						
Married	65	49.6	46	70.8	19	29.2	>0.999
Single/Divorced/Widowed	66	50.4	46	69.7	20	30.3	
**Type of contract**	N=101						
Indefinite	35	34.6	27	77.2	8	22.9	0.639
Time bound	66	65.4	47	71.2	19	28.8	
**Home province**	N=42						
Eastern province	25	59.5	17	68.0	8	32.0	>0.999
Other provinces	17	40.5	12	70.6	5	29.4	
**Duration in the hospital**	N=113						
0-2 years	53	46.9	30	56.6	23	43.4	0.019
3-5 years	38	33.6	29	76.3	9	23.7	
>5years	22	19.5	19	86.4	3	13.6	
**Ever worked outside health sector**	N=110						
No	96	87.3	63	65.6	33	34.4	0.218
Yes	14	12.7	12	85.7	2	14.3	
**Received disciplinary measures**							
No	71	50.0	46	64.8	25	35.2	0.521
Once	57	40.1	42	73.7	15	26.3	
More than once	14	9.9	9	64.3	5	35.7	

† Clinical support staff include laboratory nurses, community health nurses, hygienists, nutrionists & pharmacists;

‡ Non-clinical support staff include data collectors, social worker and monitoring & evaluation officers;

†† Column percentages;

‡‡ Row percentages

By the end of 2013, 45 (31.7%) staff left employment at Kirehe Hospital, including 9 out of 11 (81.8%) doctors and 26 out of 86 (30.2%) nurses and midwives. In the bivariate analysis, health care workers with significant attrition rates included doctors (81.8%, n=9, p=0.001), staff with bachelors level of education (50.0%, n=12, p=0.048) and health care workers employed at the hospital for up to two years (43.4%, n=23, p=0.019). In the multivariate analysis, doctors were significantly more likely to leave the district hospital relative to nurses and midwives (OR=10.0, 95% CI: 1.9-52.1, p=0.006) and staff that had up to two years at the hospital were likely to leave employment relative to those with at least five years at the hospital (OR=5.3, 95% CI: 1.3-21.7, p=0.022) (Table 2).

**Table 2:** Multivariate analysis of attrition of health care workers

Variables	OR	[95% CI]	P-value
**Type of health care worker**			
Nurses & Midwives	Ref		
Doctors	10.02	[1.92-52.12]	**0.006**
Clinical support staff^†^	1.67	[0.46-6.00]	0.435
Non-clinical support staff^‡^	0.41	[0.14-1.21]	0.106
**Duration in the hospital**			
>5years	Ref		
0-2 years	5.25	[1.27-21.67]	**0.022**
3-5 years	2.36	[0.52-10.78]	0.268
Missing	3.96	[0.86-18.31]	0.078

† Clinical support staff include laboratory nurses, community health nurses, hygienists, nutrionists & pharmacists;

‡ Non-clinical support staff include data collectors, social worker and monitoring & evaluation officers

## Discussion

Kirehe District Hospital experienced high levels of health care worker attrition in 2013, 31.7% overall and 81.8% for doctors. These rates were considerably higher than the only other study we identified exploring attrition at district hospitals in East Africa, which reported a 3.0% overall and 4.0% for doctors in Kenya [3]. While the Kenyan study might have had lower rates of attrition due to inclusion of district hospitals in both urban and rural settings, the high rural attrition rates in our study reflects the findings of a multi-country study in Africa and Asia noting low intentions of medical students to work in rural settings [12]. In a web-based survey in Rwanda, 60% of district health managers reported human resource challenges such as staff shortages and high rates of attrition as significant barriers in the health sector performance [13].

In our study, the high rates of attrition were linked to profession (doctor) and duration of employment (≤2 years). These individual factors, in addition to age, gender and marital status have been previously associated with attrition in low- and middle- income countries [1]. For high levels of doctor attrition in rural settings, factors such as high work load and burn out, poor living and working conditions, low salaries and limited career growth opportunities have been implicated [1, 11, 14].

Further, we hypothesize that the specific risk of losing doctors and individuals who have been at the hospital for a short period may be linked to the two national programs. First, the Human Resources for Health program expanded residency training opportunities for general practitioners in the same year we conducted this study [9]. While this opportunity for further training should ultimately improve the number and quality of clinicians available, it may have also contributed to the loss of doctors leaving rural areas for training in the urban settings, as similarly identified in the 2016 mid-term review of Rwanda’s third Health Sector Strategic Planning [15]. The introduction of secondary referral hospitals in each of Rwanda’s five provinces where graduate specialists from the Human Resources for Health program will be deployed to decongest tertiary referral hospitals, may improve access to this higher level care in rural areas [11]. In addition to decentralization of the specialists, strategies such as recruiting students of rural origin, including rural health needs in the training curricula and conducting training in rural facilities to increase awareness and concern for rural health challenges [1, 2, 5, 12] could improve placement and retention in the rural areas post Human Resources for Health training.

Secondly, while the compulsory service program posts general practitioners to rural district hospitals for two years of service, without interventions to encourage retention, these doctors may leave rural settings after service completion. A paper reviewing the impact of compulsory service programs globally noted that while compulsory service programs increase physician presence in rural areas in about 70 countries implementing the program, high attrition rates of physicians after service completion was a major challenge [16]. The shortage of doctors in Rwanda that leads to their high demand in urban areas and private sector, poor working conditions, and lack of opportunities for other sources of income such as in private practice make rural areas less desirable to health workers [1, 13, 14]. Recurrent attrition after compulsory service may affect the quality of care delivered, continuity of chronic care by breaking up patient-provider relationships, and the morale of remaining staff due to increased workload [10, 16]. We suggest linking compulsory service program with additional rural attraction and retention strategies such as deploying health care workers of rural origin to their home districts during compulsory service program, improving living conditions and offering professional development opportunities in the rural areas [1, 2, 5, 8].

There are a number of limitations to our study. First, since we only included one rural district hospital that receives support from Partners In Health, our study may not be generalizable to the rest of Rwanda. However, Kirehe District Hospital is similar to other district hospitals in Rwanda in terms of recruitment and management of health workers and given that the compulsory service program and Human Resources for Health program are national programs, it is reasonable to assume that other hospitals might have faced similar attrition challenges in 2013. Secondly, the individual factors we assessed were restricted to what is routinely available in the human resources files. For example, we were not able to track health worker’s origin, relocation or reason for attrition. Other factors such as health worker motivation, burn out or work load, opportunities for professional development, salaries, management and work environment are reported in the literature [1, 2, 10] and need to be studied in the context of Rwanda. In addition, strengthening the human resources for health management systems [15] by including health worker exit interviews might provide information to support analysis of the health worker attrition in the future. Finally, while Kirehe Hospital experienced high rates of health worker attrition in 2013, it is possible that through the compulsory service program or other recruitment efforts, some of the health workers who left employment in 2013 were replaced the same year. Future attrition studies should also assess replacement of health workers to give a comprehensive picture of facility staffing in a given year.

## Conclusion

The high attrition of health care workers at Kirehe District Hospital in 2013 and the general shortage of health care workers in Rwanda present challenges to ensuring rural health facilities are adequately staffed. Insufficient staffing affects quality and flow of care through high workload for health care workers. Efforts towards linking compulsory service program to not just rural placement but also retention strategies are relevant to Rwanda and other countries implementing similar programs. In addition, ensuring the future benefits of the Human Resources for Health program are equitably distributed to the rural population in Rwanda through deployment of the trained health care workers to rural settings is critical. We recommend a larger mixed methods prospective study with a longer follow-up window to assess rural health care worker needs, preferences, challenges and reasons for attrition to inform retention policies. In addition, the impact of compulsory service program and Human Resources for Health program on rural health care worker turnover and availability will improve the implementation of these programs.

### What is known about this topic

- There is a shortage and uneven geographic distribution of health workers in sub-Saharan Africa;
- Attrition drives the health worker crisis in sub-Saharan Africa;
- The rates of rural health worker attrition and individual risk factors for attrition are less commonly studied.

### What this study adds

- The rates of attrition at rural district hospital were high, with higher rates of attrition among doctors;
- The type of health worker and the health worker duration of service at a health facility were linked to attrition;
- National programs such as compulsory service program and health worker training programs might affect attrition patterns and should be evaluated.

## Competing interests

The authors declare no competing interests.

## References

[1] Lehmann U, Dieleman M, Martineau T (2008). Staffing remote rural areas in middle- and low-income countries: a literature review of attraction and retention. BMC Health Serv Res.

[2] Dolea C, Stormont L, Braichet J (2010). Evaluated strategies to increase attraction and retention of health workers in remote and rural areas. Bull World Health Organ.

[3] Chankova S, Muchiri S, Kombe G (2009). Health workforce attrition in the public sector in Kenya: a look at the reasons. Human Resources for Health.

[4] Appiagyei A, Kiriinya RN, Gross JM, Wambua DN, Oywer EO, Kamenju AK (2014). Informing the scale-up of Kenya’s nursing workforce: a mixed methods study of factors affecting pre-service training capacity and production. Human Resources for Health.

[5] World Health Organization (2010). Increasing access to health workers in remote and rural areas through improved retention: Global Policy Recommendations.

[6] World Bank (2014). World development indicators: rural environment and land use.

[7] World Health Organization (2013). Road map for scaling up the human resources for health: For improved health service delivery in the Africa region 2012-2025.

[8] Serneels P, Montalvo JG, Pettersson G, Lievens T, Butera JD, Kidanu A (2010). Who wants to work in a rural health post? The role of intrinsic motivation, rural background and faith-based institutions in Ethiopia and Rwanda. Bulletin of the World Health Organization.

[9] Binagwaho A, Kyamanywa P, Farmer PE, Nuthulaganti T, Umubyeyi B, Nyemazi JP (2013). The Human Resources for Health Program in Rwanda – A New Partnership. The New England Journal of Medicine.

[10] Buchan J (2010). Reviewing the benefits of health workforce stability. Human Resources for Health.

[11] Rwanda Ministry of Health (2012). Third Health Sector Strategic Plan: July 2012 – June 2018.

[12] Silvestri DM, Blevins M, Afzal AR, Andrews B, Derbew M, Kaur S (2014). Medical and nursing student’s intentions to work abroad or in rural areas: a cross-sectional survey in Asia and Africa. Bulletin of World Health Organization.

[13] Sayinzoga F, Bijlmakers L (2016). Drivers of improved health sector performance in Rwanda: a qualitative view from within. BMC Health Serv Res.

[14] Wurie HR, Samai M, Witter S (2016). Retention of health workers in rural Sierra Leone: findings from life histories. Human Resources for Health.

[15] Chabot J, Alebachew A, Tumusiime P, Mensah K (2015). Mid term review (MTR) of the Rwanda third health sector strategic plan (HSSP II, July 2012-June 2018).

[16] Frehywot S, Mullan F, Payne PW, Ross H (2010). Compulsory service programmes for recruiting health workers in remote and rural areas: do they work?. Bulletin of World Health Organization.

